# Gut microbiome in multiple myeloma: Mechanisms of progression and clinical applications

**DOI:** 10.3389/fimmu.2022.1058272

**Published:** 2022-12-08

**Authors:** Liuyun Zhang, Yunhui Xiang, Yanying Li, Juan Zhang

**Affiliations:** ^1^ Department of Laboratory Medicine and Sichuan Provincial Key Laboratory for Human Disease Gene Study, Sichuan Provincial People’s Hospital, University of Electronic Science and Technology of China, Chengdu, China; ^2^ School of Medicine, University of Electronic Science and Technology of China, Chengdu, China

**Keywords:** multiple myeloma, gut microbiota, hematopoietic stem cell transplantation, immune responses, vaccine immunotherapy

## Abstract

The gut commensal microbes modulate human immunity and metabolism through the production of a large number of metabolites, which act as signaling molecules and substrates of metabolic reactions in a diverse range of biological processes. There is a growing appreciation for the importance of immunometabolic mechanisms of the host-gut microbiota interactions in various malignant tumors. Emerging studies have suggested intestinal microbiota contributes to the progression of multiple myeloma. In this review, we summarized the current understanding of the gut microbiome in MM progression and treatment, and the influence of alterations in gut microbiota on treatment response and treatment-related toxicity and complications in MM patients undergoing hematopoietic stem cell transplantation (HSCT). Furthermore, we discussed the impact of gut microbiota-immune system interactions in tumor immunotherapy, focusing on tumor vaccine immunotherapy, which may be an effective approach to improve anti-myeloma efficacy.

## Introduction

Multiple myeloma (MM) is the second most common hematological malignancy with significant heterogeneity, characterized by the accumulation of malignant plasma cells in the bone marrow that in the majority of cases secrete large amounts of monoclonal immunoglobulin paraproteins (1, 2). Despite the finding of novel drugs which have improved treatment options for MM patients, curative outcomes remain elusive due to the emergence of drug resistance and relapse (3–6). Tumor immunotherapy shows great prospects in controlling or even eradicating residual disease by activating the host immune system and enhancing the anti-tumor immune effect. Vaccination of MM patients can activate the immune system and induce an immune response against the tumor antigen in the host by injecting myeloma antigens into a novel context outside the bone marrow microenvironment (BMM) (7), thereby preventing tumor growth, metastasis, and recurrence.

The intestinal microbiota plays a key role in many aspects of human physiological processes including nutrient metabolites uptake, immune regulation and response, reaction to infection (8–10), hematopoiesis, and neurobehavioural traits (11, 12). During the past few years, there is growing evidence that gut microbes also play an important role in tumorigenesis, progression, and immunotherapy of many neoplasms (13–19), particularly through interactions with the tumor microenvironment (TME). The gut microbiota modulates the tumor immune microenvironment, is involved in the initiation and development of MM as an essential contributing factor, and has been associated with immune cell recovery and treatment outcome after hematopoietic stem cell transplantation (HSCT) in myeloma patients. Recently, studies have shown that the microbiota can also impact the immune response to tumor vaccination (20, 21). Thus, targeting gut microbes and tumor vaccines to remodel and modulate TME may enhance tumor antigen specificity and immunogenicity, thereby augmenting anti-tumor immunity.

Here, we discuss the role of gut microbiota in the host immune response, summarize the gastrointestinal microbiota profile of MM patients and discuss the potential mechanisms of gut microbial alterations in myeloma progression. In addition, we summarized the correlation between gut microbiota and the outcome of HSCT in MM patients. Finally, we explored the value of clinical applications of gut microbes in MM, mainly the predictive biomarker role and the potential roles in immunotherapy with vaccination.

## Gut microbiota and immune responses

The immune system is accountable for recognizing, adapting, and responding to a myriad of foreign and self-molecules and is thereby vital in both pathological and physiological conditions (22). Numerous studies have revealed that communication between the gut microbiota and the host is essential for the development of the innate and adaptive immune system (23, 24), particularly to support the development and function of immune cells, including macrophages, B cells, T cells, dendritic cells (DCs), and neutrophils, which in turn shape the microbial structure (25, 26). Antigen-presenting cells (APCs) such as DCs and macrophages in the lamina propria can integrate microbial signals to maintain gastrointestinal homeostasis and adaptive immunity (27). Antigens of commensal microbial origin can be recognized by APCs, and antigens can stimulate lymphocytes, macrophages, and DCs in the lamina propria, which together produce a series of extrinsic stimuli that play a key role in host immunity and metabolism (27, 28). One of the first immunologic deficiencies noted in germ-free mice was a significant deficiency in secretory immunoglobulin a (sIgA) levels in the intestine (29). Additionally, excessive activation of anti-inflammatory Th2 cytokines, decreased expression of MHC class II, and reduced expression of pattern recognition receptors, such as toll-like receptors, were also observed in germ-free animals (30). Furthermore, impaired basal Stat1 signaling and altered T-cell homeostasis were found in mice with gut microbial depletion induced by treatment with broad-spectrum antibiotics and resulted in impaired progenitor cell maintenance and granulocyte maturation (31), which injured hematopoiesis. These findings clearly indicate that gut microbes are essential in host immune regulation.

It is well known that intestinal commensals can maintain the epithelial barrier and intestinal integrity by synthesizing and releasing a myriad of metabolites. These metabolites can enter the circulation and can act as metabolic substrates or induce the production of inflammatory cytokines to exert immunomodulation and influence the immune response (32). For example, succinate and aconitate, which are known to be by-products of microbial fermentation, can modulate the functional phenotype of mouse bone marrow-derived macrophages in response to bacterial infection (33). In addition, microbial-derived metabolites are absorbed in the host gut and enter the host circulation, where they can often reach or exceed the concentration of the therapeutic drug (34). Many studies have found that microbial-derived metabolites, such as short-chain fatty acids (SCFA), can exert anti-inflammatory effects through various downstream signaling pathways thereby promoting the inhibition of multiple tumors and controlling tumor growth (35–37). Therefore, modulation of the intestinal flora and upregulation of beneficial gut microbial metabolites may enhance the antitumor immune response.

## Gut microbiota in MM and possible mechanisms in MM development

MM oncogenesis is a complex process, impacted by genetic and environmental factors with various pathological mechanisms. Although gene expression programs seem to be driven by primary genetic events, there is a sequence of molecular events that are caused by these abnormalities, including secondary genetic abnormalities, as well as genetic events of monoclonal gammopathy of undetermined significance (MGUS) and MM progression such as aberrant nuclear factor-κB (NF-κB) signaling (38). In addition, MM development and tumor cell resistance are also closely linked to their BMM. Some of these mechanisms, including chronic antigenic stimulation of B-cells, inflammation, and immune modulation, are closely connected to the intestinal microbes and have been extensively reviewed (25, 39, 40). Here, we highlight specific microbial-related components in MM tumorigenesis and progression that are associated with potential regulation and translation for clinical therapy.

**Figure 1:**
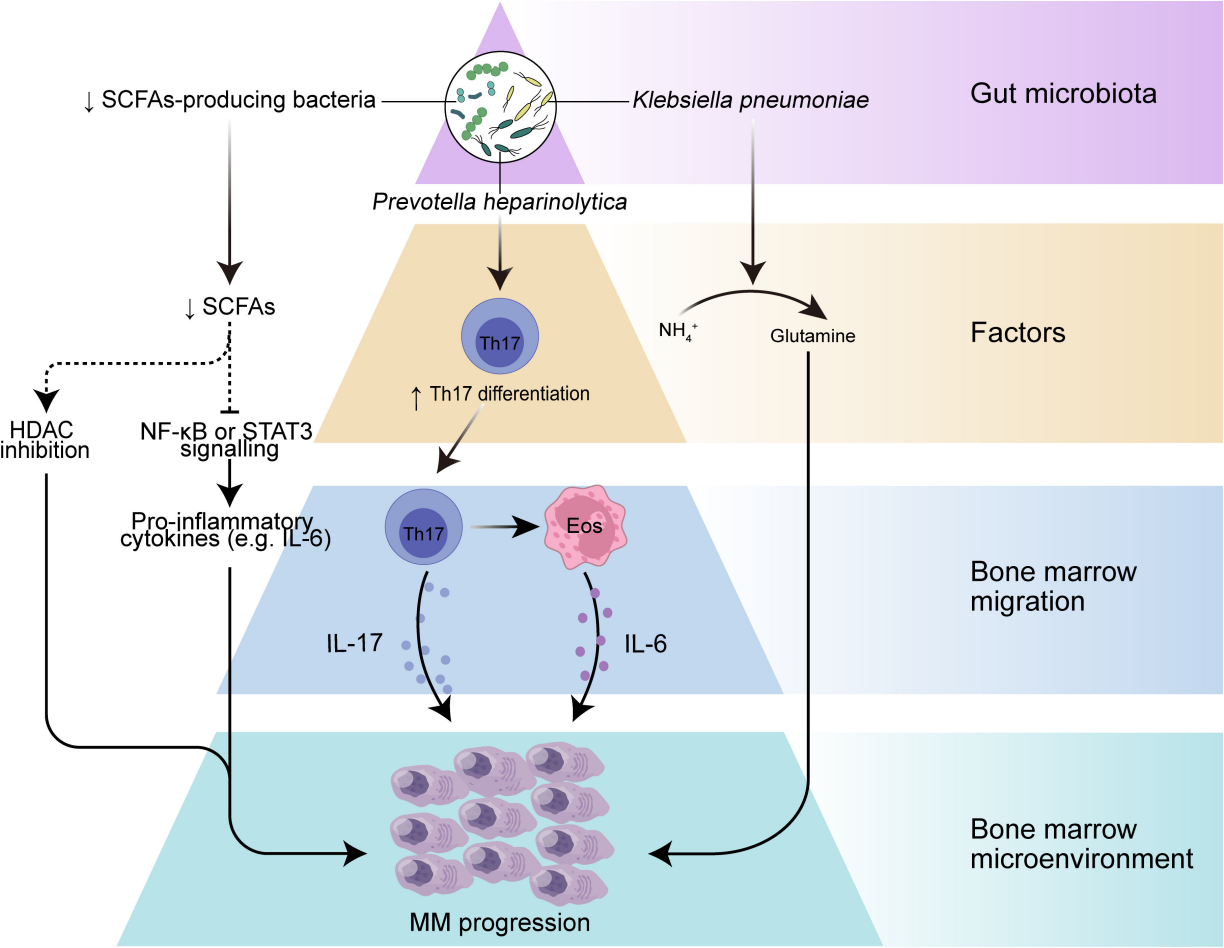
The role of gut microbiota in myeloma progression. Dysbiosis of the gut microbiota leads to a reduction in the microbiota-producing SCFAs, which can result in decreased levels of SCFAs, leading to increased activation of inflammatory mediators such as IL-6 and NF-κB pathways, and may further drive myeloma progression. A selective increase in nitrogen-recycling microbiota in the intestinal lumen may promote glutamine synthesis and lead to myeloma progression. In addition, specific bacteria such as *Prevotella heparinolytica* promote intestinal epithelial colonization and bone marrow (BM) migration of Th 17 cells in the Vk*MYC mouse model, which can lead to the production of IL-17 by Th 17 cells in the BM and further activate eosinophils and promote IL-6 release, thereby contributing to inflammation-mediated tumor progression.

Early evidence from mouse models favored a role for microbes in MM development. The most abundant components of the human intestinal microbiota are bacteria that mainly belong to four phyla, namely *Firmicutes*, *Bacteroidetes*, *Actinobacteria*, and *Proteobacteria* (41), and the role of gut microbes in humans is usually studied using metagenomic sequencing or 16S ribosomal RNA (rRNA) sequencing. The MM microbiota exhibits compositional alterations (usually termed microbiota dysbiosis) compared with the gut microbiota of healthy individuals, reflecting a different ecological microenvironment in MM patients (19, 42). The abundance of *Proteobacteria* was found to be increased in MM subjects, while the abundance of *Actinobacteria* was reduced at the phylum level. In addition, *Streptococcus*, *Klebsiella*, *Clostridium leptum*, and *Pseudomonas aeruginosa*, as well as bacteria from *Odoribacter* and *Lactobacillus* showed increased abundance in stool samples from MM patients. These bacterial disturbances may lead to changes in microbiota-derived metabolites that function as signaling molecules and substrates for immune and metabolic responses and thus may contribute to the progression of MM (**Figure 1**).

### Inflammation and immune regulation

The gastrointestinal tract provides an important site for crosstalk between the microorganisms and the host immune system (30). Intestinal DCs can sense intestinal colonized bacteria and present their antigens in mesenteric lymph nodes or Peyer’s patches, and further, DCs are responsible for the differentiation of T cells in lymph nodes into regulatory T cell (Treg), T cell 17 (Th17), Th1 and Th2 cells and the production of pro- or anti-inflammatory cytokine profiles (43, 44). The close interaction of the gut microbiota with the host immune system influences not only host immune regulation but even the inflammatory environment of tumor progression. A prominent example is that excessive inflammation of Th17 cells or excessive immunosuppression induced by Treg may promote development of cancer (45). Differentiation of intestinal Th17 cells contributes to intestinal mucosal barrier protection from harmful pathogens and in turn, commensal bacteria from the gut are involved in the differentiation of Th17 cells (46), which are characterized by the production of interleukin-17A (IL-17A), IL-17F and IL-22. These cytokines play a key role in inflammation, and IL-17 among them can promote tumor progression by affecting the TME (47, 48). Calcinotto et al. provide evidence that gut-specific bacteria can promote cancer progression using a mouse model of transgenic VK*MYC mimicking human MM (49). They found that *Prevotella heparinolytica* promoted the differentiation of Th17 cells colonizing the intestine of VK*MYC mice and entering the BM of transgenic mice, where they favored MM progression. IL-17 not only acts directly on IL-17R-expressing tumor plasma cells but also modifies BMM through local activation of eosinophils and thus exerts a pro-tumor effect. This study revealed an immune axis between IL-17 and eosinophils, specifically, IL-17 stimulates eosinophils and induces them to produce tumor necrosis factor-α (TNF-α and IL-6. This axis can be broken by a combination of cytokine-specific antibodies (monoclonal antibodies targeting IL-17RA, IL-17A and IL-5), showing a promising therapeutic strategy. In conclusion, these results suggest that specific bacteria may directly influence the migration of immune cells and the secretion of inflammatory cytotoxic factors and play an important role in tumor progression.

### Metabolism of gut microbiota-derived metabolites: SCFA and L-glutamine

Metabolism is another key area of interaction between the host and microbiota. The microbial metagenome encodes genes that metabolize many dietary nutrients, including dietary fiber and host compounds (50). SCFAs represent the main class of metabolites produced during the fermentation of insoluble dietary fiber by intestinal microbiota (51), principally including butyrate, propionate, and acetate (52), and play key roles in intestinal regulation and immune modulation through modulating signaling pathways (53, 54). Butyrate is considered an anti-inflammatory molecule that can inhibit lipopolysaccharide (LPS)-stimulated neutrophil production of pro-inflammatory cytokines, such as TNF-α and cytokine-induced neutrophil chelator-2, as well as nitric oxide, and can attenuate LPS-activated NF-κB (36). Butyrate can also inhibit histone deacetylases (HDAC) in colonocytes and immune cells, thereby reducing pro-inflammatory cytokines (55). MM patients had significantly reduced levels of *Anaerostipes hadrus, Clostridium butyricum*, and *Clostridium saccharobutylicum*, which are the main SCFAs-producing microorganisms, compared to healthy controls (19, 56, 57). Furthermore, remission of tumor progression was observed in a MM mouse model with the addition of *Clostridium butyricum* (19), a common butyric acid-producing bacterial species that has been shown to control colorectal cancer progression by inhibiting the Wnt/β-catenin signaling pathway and modulating the gut commensal composition (37). These findings suggest that SCFAs from the gut microbiota may play an important role in MM progression. Despite the specific mechanisms underlying the role of SCFAs in MM being unclear, SCFAs often exert tumor suppressive effects by downregulating the NF-κB pathway, inhibiting inflammatory factors such as IL-1β, IL-6, IL-8, and TNF-α, and inducing the expression of IL-10 anti-inflammatory cytokines. Many studies have demonstrated that these high levels of inflammatory mediators in the MM bone marrow microenvironment greatly contribute to MM progression. Although normally considered a tumor suppressor metabolite, the deleterious effect of SCFAs had been reported to promote the growth of prostate cancer in mice on a high-fat diet (58). This apparent contradiction may be due to its heterogeneous influences on the dissimilar genetic background and dietary habits. Nevertheless, the exact mechanism of how SCFAs affect MM deserves further investigation, and it is possible that increasing the abundance of SCFAs-producing gut microbiota could inhibit the secretion of inflammatory factors and activation of NF-κB signaling pathways to control inflammation and further slow the progression of MM.

L-glutamine (Gln) represents another metabolite related to gut microbiota. Glutamine is a nutrient that can be used as a carbon or nitrogen source to support intracellular energy production and biomass synthesis, and it can also serve as a nitrogen donor to meet the increased demand for nucleotide biosynthesis in cancer cells to support cancer growth (59, 60). To supply the metabolic requirements of Gln, mammalian cells are dependent on glutamine synthetase, an enzyme that gains Gln from glutamate (Glu) and ammonium ( ${\text{NH}}_{4}^{+}$ ) (61). MM cell metabolism can consume large amounts of Gln and may produce excess ${\text{NH}}_{4}^{+}$ , in addition, MM cell growth is limited by Gln depletion (61, 62). Fecal samples from MM patients were significantly enriched in bacteria associated with nitrogen use and recycling (e.g. *Klebsiella* and *Streptococcus*), compared to the healthy population (19). Researchers further transplanted *Klebsiella pneumoniae* into mice with MM and found that *Klebsiella pneumoniae* contributed to the progression of MM through *de novo* synthesis of glutamine. In addition, they observed a significant increase in Gln concentration in the serum and feces of mice treated with ${\text{NH}}_{4}^{+}$ or urea and found that accumulation of abnormal amino acids and blood urea in the host BMM could promote the proliferation of nitrogen-cycling microbiota, which in turn could facilitate urea degradation and glutamine synthesis (19). These results suggest that gut microbes can provide MM cells with a nitrogen source to meet their energy requirements for Gln synthesis and rapid proliferation, thereby accelerating MM progression.

## Role of Gut microbiota in HSCT for MM patients

### Gut microbiota and auto-HSCT in MM

Several observational studies in patients with hematologic malignancies have established that different HSCT outcomes such as risk of death, recurrence/progression of disease after transplantation and transplantation-related adverse events are largely associated with the integrity of gut microbes or the abundance of certain bacteria (63–65). Generally, HSCT results in a loss of gut microbial diversity, but higher gut microbial diversity before or during transplantation predicts better transplantation outcomes.

Newly diagnosed MM patients are highly sensitive to first-line therapy and high-dose chemotherapy with autologous HSCT (auto-HSCT) is an important treatment regimen for MM patients (66). However, chemotherapy used for auto-HCT often damage the intestinal mucosa and contribute to significant changes in intestinal microbiota structure. D’Angelo et al. conducted a single-center, small-sample prospective study assessing changes in the gut microbiome for MM patients in first year following auto-HSCT transplantation and found that reduced gut microbial alpha diversity at the time of cell implantation was associated with impaired patient response to auto-HSCT (67). Moreover, patients treated with auto-HSCT with lymphoma, MM, and amyloidosis had lower fecal microbiota diversity in the early pre-transplant period and further decreased during transplantation compared to healthy controls, and in addition, MM patients with higher diversity had a lower risk of disease progression or death during the peri-neutrophil transplantation period (68). Indeed, the composition of the gut microbiota of MM patients at baseline was also associated with the incidence and severity of post-transplant nausea, vomiting, and neutropenia, as well as with the rate of neutrophil implantation (63). These results suggest that the structural integrity of intestinal microbiota plays an essential function in facilitating a good prognosis after HSCT.

HSCT-related events and patient immunity are also associated with specific alterations in the gut microbiome. A higher abundance of fecal *Bacteroidetes* was correlated with milder gastrointestinal symptoms, whereas enrichment in *Blautia* and *Ruminococcus* was correlated with a high incidence of diarrhea and post-transplant nausea and vomiting. The researchers also found that a high abundance of beneficial bacteria such as butyrate-producing *Eubacterium hallii* and *Faecalibacterium prausnitzii* were implicated in minimal residual disease (MRD) negativity (69). Specific bacterial changes may also indicate reduced immune function in patients. For example, butyrate-producing *Bifidobacteria* and genera, a beneficial bacterium, are significantly reduced in pre-allo-HSCT patients, and these bacteria have been reported to inhibit the worsening of intestinal inflammation and their product butyrate is one of the substances required to maintain normal mucosal function and regulate local immune responses through the induction of Treg (70). Although the exact mechanism of how microbes act on graft outcome remains unclear, the above results support the beneficial role of gut microbes in treatment, such as protection of the intestinal mucosa. Therefore, correcting the gut microbiota and modulating the immune system to rebuild the immunity of MM patients may be a new approach to improve treatment outcomes and reduce drug toxicity in MM patients. The current studies on the correlation between HSCT and gut microbiota in MM are mostly single-center and small samples, which have significant limitations and therefore need to be optimized and validated in larger cohorts.

### Gut microbiota and HSCT-related BSI in MM

In patients treated with HSCT, the associated disease and the use of therapeutic drugs or antibiotics (ATB) often destroy the gastrointestinal mucosal barrier and the stability of intestinal flora structure, usually characterized by lower microbial diversity and a decrease in beneficial intestinal commensal bacteria or an increase in opportunistic pathogens. In patients with low intestinal microbial diversity, the intestinal flora is typically dominated by a single genus, including *Streptococcus*, *Enterococcus*, *Lactobacillus*, *Kluyvera*, *Klebsiella*, *Staphylococcus*, and *Escherichia* (65, 71). The bacterial (mainly *Enterococcus*, *Streptococcus*, and various *Proteobacteria*) dominance has been shown to be associated with subsequent bacteremia during allo-HSCT, and *Enterococcal* dominance led to a 9-fold increased risk of Vancomycin-resistant *Enterococcus* bacteremia, while *Proteobacteria* domination led to 5-fold increased risk of gram-negative rod bacteremia (72). The increased susceptibility to bloodstream infection (BSI) is largely related to impaired immune system and inability to resist pathogens caused by the destruction of intestinal microbiota. An animal experiment has confirmed that intestinal microbial destruction can suppress the immune system, with evidence suggested that mice with microbiota depletion caused by broad-spectrum antibiotic treatment have altered T-cell homeostasis and impaired progenitor cell maintenance and granulocyte maturation (31). Additionally, antibiotic exposure typically leads to gut microbiome disturbances and is associated with impaired survival outcomes in HSCT. Interestingly, researchers found that exposure to levofloxacin was associated with a trend toward a reduced risk of predominance of non-*Bacteroidetes* genera and found that levofloxacin for BSI prophylaxis was less damaging to the gut microbiota of patients with acute myeloid leukemia or MM than broad-spectrum β-lactams, which are commonly used to treat neutropenic fever (73), suggesting that there are differences in the degree of damage to gut microbes by different antibiotics. In MM patients, initiation of levofloxacin prophylaxis was related to a non-significant increase in the risk of bacteremia caused by levofloxacin-resistant *Enterobacteriaceae* and *Clostridium difficile* (*C. difficile*) infection (74). Excitingly, fecal microbiota transplantation (FMT) has been reported to be effective in the treatment of recurrent *C. difficile* infection (75), a common infection after HSCT that is associated with a significant incidence rate and mortality, especially early post-transplantation (76). The above results suggest that the composition of the gut microbiome plays an important role in the prevention of complications after HSCT and that modulating the composition of the gut microbes may ameliorate these adverse effects and further show that gut microbes play an important role in the recovery of host immunity.

## Challenges of using microbiota as a biomarker for MM patients

A new clinical application of the gut microbiota is as a biomarker to screen for disease or assess therapeutic efficacy and clinical outcomes. However, there are many challenges in utilizing gut microbes as a biomarker. Gut microbes used as tumor screening markers need to have a significant disease relevance (e.g., *Helicobacter Pylori* and gastric cancer) or an identifiable characteristic structure, preferably with characteristic alterations that can be observed in antecedent disease. It is well established that MM develops through gradual evolution, usually from an asymptomatic precursor entity, such as MGUS or smoldering multiple myeloma (SMM), to transform and develop into symptomatic MM (77). Current microbiota data on comparing MM patients with healthy controls and precursor disease is not all published and is limited by studies with small numbers of patients. Moreover, microbiome data are generally not readily available in real-time in the clinic and therefore are usually retrospective and cannot currently be used to predict future clinical events. A great challenge is how to obtain prospective microbiome data during disease process and turn them into markers to predict important clinical events.

From current studies, gut microbiome has great potential in predicting the prognosis of myeloma treatment. Several studies have reported a strong association between gut microbial diversity and clinical outcomes and complications (63, 68). In patients with myeloma, MRD negativity is associated with the abundance of butyrate-producing microbiota such as *Eubacterium hallii* and *Faecalibacterium prausnitzii* (69). Consistently, there is an association between sustained MRD negativity and fecal butyrate (78). This also highlights the potential of butyrate-producing bacteria as markers in MM patients. In addition, pneumonia is an important cause of morbidity and mortality in MM patients. A recent study found that enriched *Klebsiella pneumoniae* was associated with the incidence of pneumonia in MM by the mechanism that intestinal colonized *Klebsiella pneumoniae* indirectly causes pneumonia in MM through glutamine synthesis (79). However, most of these studies focused on HSCT and were mostly small samples and from a single center, or did not separately discuss the correlation between gut microbes and myeloma subgroups. Further exploration of the association between bacterial biomarkers and clinical outcomes in MM in larger and multicenter cohorts might improve the accuracy of gut microbes as prognostic markers. Researchers have also searched for microbial biomarkers beyond stool samples. One study reported that oral microbiota characterization could reflect the adverse effects of bisphosphonate therapy, with findings indicating that reduced bacterial abundance or diversity was associated with bisphosphonate-related osteonecrosis of the jaw (BRONJ) in patients with MM (80). In summary, microbiota structure and specific taxa have great potential as predictive markers for therapeutic response in MM patients, but more data on the genomic characteristics of the gut microbes in MM patients are needed before they can be applied in the clinic.

Of note, the commonly used method for gut microbial detection, 16S rRNA sequencing, only identifies bacteria, which results in a lack of information on fungi or viruses. The use of 16S/18S/ITS rRNA sequencing or metagenome shotgun sequencing or metatranscriptomics sequencing will yield more relevant information, such as key species, metabolic signatures and metabolic pathways. Also, sequencing data combined with metaproteomics or metabolomics data can reveal disease-associated active substances and predict changes in disease-associated metabolic pathways. In MM, measurement of SCFA levels (especially butyrate) in stool and serum by targeted metabolomics may improve the ability to predict clinical outcomes. However, more studies are needed to explore whether biomarkers from the microbiome will have clinical relevance in MM.

## Harnessing the gut microbiota for MM immunotherapy

**Figure 2:**
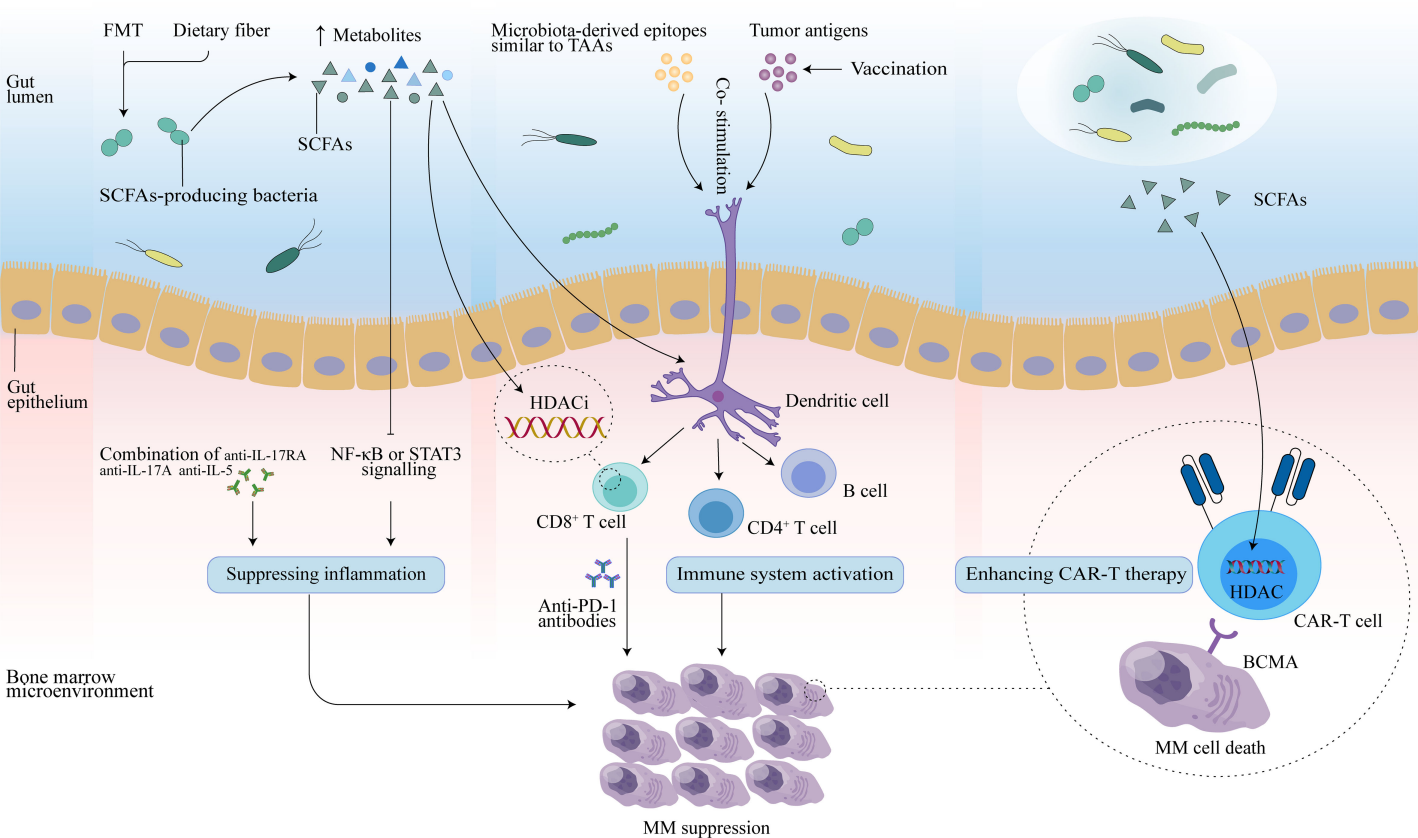
Potential therapeutic effects of the gut microbiota in MM. FMT may reconstruct the gut microbiota, and dietary fiber may increase levels of SCFAs secreted by SCFAs-producing microbiota. Butyrate in SCFAs may promote CD8^+^ T function by inhibiting HDAC, and activated CD8^+^ T cells combined with anti-PD-1 antibodies may enhance anti-myeloma effects. In addition, SCFAs can slow MM progression by inhibiting Th17 cells, and further reducing inflammatory cytokines (IL-17 and IL-6) that promote MM progression. Combination therapy with anti-IL-17RA, anti-IL-17A, and anti-IL-5 may also mediate antitumor effect. Vaccination controls tumor growth by activating T- and B-cell-mediated immunity through activation of the antigen-presenting capacity of DCs, thereby inducing an immune response against tumor antigens in the host. Microbial epitopes similar to tumor-associated-antigens (TAAs) may interact with DCs to enhance the vaccine response. Additionally, SCFAs may also enhance anti-tumor activity by reprogramming CAR-T cells through HDAC.

Immunotherapy, which relies mainly on immune cells inside or outside the tumor microenvironment to specifically recognize and attack tumor cells, is an effective treatment for many cancers. It is well known that the interaction of antigenic components of the gut microbiota and the products of their metabolic activity with host cells affects the immune response and can promote immunomodulatory effects and a variety of tumor-suppressive effects (81). In addition to its role in oncogenesis and tumor progression, there is growing evidence that the gut microbiota plays a role in the efficacy and toxicity of immunotherapy (20, 82, 83). This allows the gut microbiome to be used as a biomarker to forecast therapeutic response or adverse effects, as well as to be modulated to improve tumor immunotherapy and patient prognosis (**Figure 2**).

### Gut microbiota for MM vaccine immunotherapy

Cancer vaccines show great potential for the immunotherapy of malignant tumors due to their low toxicity and sustained immune intervention. Cancer vaccines mainly use specialized antigen-presenting cells (e.g., DCs) to recognize antigenic substances such as tumor cells or tumor-associated antigens (TAAs) or peptides and activate naïve antigen-specific T cells to induce a specific and persistent immune response to tumor antigens in the host, thereby specifically clearing minimal residual tumor lesions or significantly inhibiting tumor proliferation and preventing cancer recurrence (7, 84). However, the highly individual heterogeneous immunotherapeutic response to tumor vaccines and how to trigger a strong anti-tumor immune response in cancer patients remains a great challenge. Intriguingly, the gut microbiota, which is also significantly individualized, appears to correlate with the efficacy of immunotherapy with tumor vaccines (85).

Cancer vaccines consist of four key active ingredients, namely, tumor antigens, formulation, immune adjuvant, and delivery vehicles. Ragone et al. demonstrated for the first time that TAA share homology with peptides derived from *Firmicutes* and *Bacteroidetes* phyla in linear sequence as well as in structure and conformation, which together account for 90% of the intestinal microbiome, including melanoma antigen-C1/cancer testis antigen 7 (MAGE-C1/CT7), MAGE-A3, and MAGE-A10 (86). In general, TAAs are characterized by low immunogenicity and poor anti-tumor effect due to different expression levels in normal cells, while gut microbiota antigens are non-self and have strong immunogenicity (87). Therefore, highly similar antigenic epitopes between TAAs and microbial antigens are very likely to induce a strong cross-reacting CD8^+^ T cell response, specifically, T cell memory induced by specific microbial antigens may turn out an anti-cancer T cell memory, thereby controlling cancer growth in the long term. Evidences that T cells induced by specific microbial antigens are able to cross-react with specific homologous TAAs have been reported, such as molecular mimicry between MAGE-A10 and cytomegalovirus (88). Indeed, two TAAs, MAGE-A3 and MAGE-C1, are also overexpressed in MM patients. Additionally, these two antigens, along with other MM-associated antigens, including Wilms tumor 1 (WT1), MM special antigen 1 (MMSA-1), and Dickkopf-1 (DKK1), have been developed as tumor vaccines and used in combination with DCs vaccination or other strategies for immune vaccination for MM patients (89–91). This helps to explore the cross-reactivity between myeloma-associated TAAs and microbial antigens, and provides new ideas for improving the efficacy of myeloma vaccine therapy. In summary, molecular mimicry between gut microbial antigens and tumor antigens induces cross-reactivity, and may facilitate the formation of a novel preventive anti-cancer vaccine strategy. Furthermore, due to some limitations of TAAs, such as induction of auto-immune reactivity, further exploration of molecular mimicry between tumor-specific antigens (TSAs) and microbial-derived antigens for the development of tumor vaccines is valuable.

In addition to molecular mimicry between bacteria and TAAs, the influence of the gut microbiota on the anti-cancer immune response to vaccines can also be driven by the regulatory effects of the gut microbiota on the host immune system and on the development and differentiation of immune cells. Most tumor vaccines enhance the immune response by stimulating APCs to present tumor antigens and thus exert anti-tumor effects, among which DCs are the most potent APCs in the immune system. Radojević et al. reported a significant correlation between the composition of the gut microbiota and the immunogenicity of DCs by analyzing the fecal microbiota composition of 14 healthy donors, together with the phenotype and cytokines produced by monocyte-derived DCs (92). Donors with high levels of α-diversity and high abundance of SCFA-producing bacteria and SCFAs showed lower CD1a expression in immature (im)DCs and higher expression levels of ILT-3, co-stimulatory molecules (CD86, CD40) pro-inflammatory cytokines (TNF-α, IL-6, IL-8), and these changes related to their lower maturation potential and immunogenic properties upon LPS/interferon gamma (IFN-γ) stimulation. Conversely, imDC derived from donors possessing lower α-diversity and higher relative abundance of *Bifidobacterium* and *Collinsella* exhibited higher CD1a expression and elevated potential to upregulate CD86 and CD40 expression, and the potential to increase TNF-α, IL-6, and IL-8 production. These results suggest that the gut microbiota plays an important role in the differentiation of donor precursor cells into immunogenic DCs suitable for cancer therapy and may enhance antitumor therapy by modulating gut microbes to optimize DC vaccines for enhanced dendritic cell function and activation of cytotoxic CD8^+^ T cells (20, 92).

Notably, most tumor vaccines alone do not directly eliminate tumor lesions, but tumor vaccines are promising in eliminating MRD. In MM, vaccines are often considered to have the greatest potential in combination with auto-HSCT or other effective therapies with immunomodulatory mechanisms of action, such as MM GM-CSF-secreting vaccine in combination with lenalidomide, and the vaccines against PD-L1 (93, 94). Researchers found that mRNA electroporation of three types of tumor-associated antigens (TAAs)-mRNA-loaded Langerhans dendritic cell vaccines (i.e., electroporated CT7, MAGE-A3, and WT1 mRNA) showed good tolerability in MM patients undergoing auto-HSCT and found that after vaccination, and antigen-specific CD4^+^ T and CD8^+^ T cells secreted pro-inflammatory cytokines such as IFN-γ, IL-2, and TNF-α were significantly increased, as well as a trend towards the increased clonal expansion of CD4^+^ T and CD8^+^ T cells, and although significant differences were not shown, probably due to limited sample size, DC vaccine-based therapy induced antigen-specific immunity after auto-HSCT, showing great promise for anti-myeloma immunity (89). This promise may be based on the fact that the lymphocytic phase in myeloma patients after myeloablative chemotherapy and their own hematopoietic stem cell transplantation provides a favorable environment for the generation of antitumor immunity through vaccination (90). A preclinical study observed that the reduction in gut microbiota diversity induced by an antibiotic cocktail was associated with an enhanced intra-tumor-specific immune response produced by the injection of a neoantigen cancer vaccine (20). They also observed that in vaccinated mice, ATB administration promoted alterations in the composition of several bacteria, including a decrease in the relative distribution of strains such as *Lachnospiraceae* involved in Treg development and an increase in *Bacillales* (gen. *Bacillus*) and *Alphaproteobacteria* (gen. *Bosea*) associated with amino acid metabolism. This suggests that treatment-induced ecological dysregulation plays an important role in the prevention of vaccine-induced immune responses. Similarly, as mentioned previously, the gut microbial composition of MM patients receiving HSCT is disrupted. Therefore, this favorable environment for vaccine action, in addition to the release of tumor antigens due to cell death (95), may also be associated with the transfer of intestinal flora and other microbiota leading to more toll-like receptor agonists and thus enhanced dendritic cell activation (96). These results reveal an intricate interplay between the microbiota, immune system, and tumor vaccine, which may play an essential role in regulating myeloma vaccine immunotherapy and may suggest that interventions based on gut microbial immunomodulation combined with vaccination are expected to improve the efficiency and intensity of the vaccine antitumor immune response or prolong the duration of the immune response, especially for vaccination after auto-HSCT is expected to clear trace residual tumor cells in patients, prevent recurrence, and improve clinical outcomes. However, more clinical validation is still needed.

### Monoclonal antibodies

The migration of Th17 cells influenced by intestinal microbes is involved in the pathogenesis of MM mice (49). It was also found that the combination of three monoclonal antibodies, namely anti-IL-17RA, anti-IL-17A, and anti-IL-5, significantly slowed disease progression in VK*MYC mice, and this slowing was associated with a reduced accumulation of Th17 cells and eosinophils in the bone marrow. In addition, anti-17A monoclonal antibodies and anti-IL5 antibodies have been approved for the treatment of immunomodulatory diseases such as psoriasis (97) and eosinophilic asthma (98). Therefore, monoclonal antibodies which target inflammatory pathways modulated by microbiota which affect MM biology or pathogenesis could be a potential therapeutic modality.

### Immune checkpoint inhibitors

Immune checkpoint inhibitors (ICIs), such as therapeutic blockade of PD-1 and PD-L1, have been shown to be an effective treatment for a variety of solid tumors or hematologic malignancies. Plasma cells in MM patients highly express PD-L1 and single-agent PD-1 blockers have a favorable safety profile, however, there is no single-agent activity for MM patients (99). Moreover, PD-1 blockade combined with existing therapies drugs such as immunomodulatory drugs (IMiDS) and protease inhibitor (PI) not only did not show an improvement in disease response for MM patients, but also resulted in increased toxicity or mortality (99, 100). Recent studies have demonstrated that intestinal microbiota can affect the therapeutic response to ICI and related toxicity (101–103). The gut microbial metabolite butyrate may modulate the expression of ID2, a key transcriptional regulator of immune cell differentiation and activation, through its HDAC inhibitory activity, thereby directly promoting the anticancer effects of CD8^+^ T cells (104) and improving the efficacy of anti-PD-L1 antibodies. Indeed, the abundance of SCFAs-producing bacteria was significantly reduced in MM patients (19). It is promising to improve the anti-PD-1 immunotherapy response in MM patients by modulating the gut microbial structure with strategies such as FMT, dietary fiber, antibiotics, prebiotics, probiotics, or postbiotics (105, 106) to increase butyrate production and thus enhance the antitumor effect of CD8^+^ T cells. Whether the combination of modulation of microbiota and immune checkpoint blockade could benefit MM patients is a question worthy of further study.

Other immune checkpoints such as LAG3 and TIGIT have also been found to be highly expressed on T cells in MM patients, which can cause immune effector dysfunction. Preclinical studies have shown that targeting TIGIT or LAG3 can restore CD8^+^ T cell immunity, thereby overcoming immunosuppression and enhancing anti-myeloma immunity (107, 108). The expression of T-cell surface immune checkpoint markers such as Tim3, Lag3 and especially PD1 has been reported to be upregulated by polysaccharide A (PSA), which is a capsular carbohydrate from the symbiotic intestinal bacteria *Bacteroides fragilis* (109). This finding contributes to a better understanding of the link between commensal microbes and immune checkpoints, but more research is needed on the role of gut microbes in combination with immune checkpoints in tumors.

### CAR-T therapy

Chimeric antigen receptor (CAR) T-cell therapy, as one of the most important therapeutic strategies for hematological malignancies, has attracted extensive attention in recent years. However, due to individual patient characteristics, the efficiency and associated immune-related adverse effects of CAR-T therapy vary considerably among patients. The gut microbial composition exhibits great individual specificity (110), which may be an important environmental factor contributing to individual differences in response to treatment. Intriguingly, recent studies have revealed a potential role for the gut microbiota in the efficacy and toxicity of CAR-T cell therapy for B-cell-derived hematologic malignancies, including relapsed/refractory (r/r) MM, acute lymphoblastic leukemia (B-ALL), and non-Hodgkin lymphoma (B-NHL) (111, 112). Hu et al. have reported that B-cell maturation antigen (BCMA) CAR-T cell therapy-related cytokine release syndrome (CRS) and treatment response is regulated by the intestinal microbiome in MM (111). Taxonomic analysis by bacterial 16S rRNA gene sequencing of the fecal microbiota showed that high abundance of genus *Sutterella* was related to both complete remission (CR) and prolonged survival after CAR-T treatment, in addition, *Bifidobacterium* and *Leuconostoc* were found to be abundant in MM patients with severe CRS while *Butyricicoccus* were abundant in patients with mild CRS (111). *Bifidobacterium* is a probiotic commonly used to boost host immunity, and its species composition can influence cytokine production (113). Thus, *Bifidobacterium* enriched in patients with severe CRS in CAR-T therapy may consist of more diverse strains of *Bifidobacterium* susceptible to CRS, while the species and strain composition of *Bifidobacterium* and the relationship with CRS need further investigated.

CAR-T therapy involves isolating T cells from a patient’s blood, genetically modifying them to express CAR receptors on their surface, and reinjecting them into the patient so that they can increase the immune response (114). Intestinal microbiota, as an environmental factor affecting immune system function, may impact the efficacy of CAR-T therapy. Bioactive metabolites released by the intestinal microbial community, such as amino acids, SCFAs, and bile acids, play significant immunomodulatory functions. Microbial derived SCFAs pentanoate and butyrate have been reported to enhance the antitumor activity of CAR-T cells through metabolic and epigenetic reprogramming (115). In addition, researchers found that pathways associated with amino acid metabolism, including phenylalanine, glutamate, tyrosine and arginine metabolism pathway, were differentially abundant across treatment outcomes and CRS grades in MM patients treated with CAR-T (111). These amino acids play an important role in the regulation of immune response by stimulating T lymphocytes, B lymphocytes and natural killer cells, regulating gene expression and lymphocyte proliferation, and promoting the production of antibodies, cytokines and other cytotoxic substances (116). Taken together, these findings may indicate that specific gut microbiota structures and derived metabolites have the potential to optimize CAR-T therapy for human tumors.

## Conclusion

In conclusion, the gut commensal microbiota is critical in regulating the function of the immune system, and several studies have shown a significant role for the gut microbiota in MM pathogenesis and progression by influencing inflammatory pathways and host metabolism. Gut microbial metabolites can influence host immunity, and in particular, the most studied SCFAs may significantly influence the outcome for patients with MM. After auto-HSCT, higher gut microbiota diversity linked with better outcome, which may be a rationale for avoiding injury to the microbiota through judicious use of broad-spectrum antibiotics. Recent studies suggest that gut microbes may influence vaccine efficacy, including the ability of antigens to elicit protective immune responses and the ability of the immune system to respond properly to antigenic stimulation, by acting as immunomodulators and natural vaccine adjuvants. Thus, it seems highly probable that there is a role of the gut microbiota in combination with vaccine immunotherapy for MRD in MM after receiving HSCT. Despite the important role of the microbiota in modulating the vaccine immune response, further investigations are needed to definitively demonstrate this possibility.

## Author contributions

LZ wrote and reviewed the manuscript. YX, YL, and JZ reviewed and proofread the manuscript. All four authors provided substantial contributions to this review, drafted and critically revised the manuscript, designed and created figures. All authors contributed to the article and approved the submitted version.
